# Linking microbial contamination to food spoilage and food waste: the role of smart packaging, spoilage risk assessments, and date labeling

**DOI:** 10.3389/fmicb.2023.1198124

**Published:** 2023-06-22

**Authors:** Shraddha Karanth, Shuyi Feng, Debasmita Patra, Abani K. Pradhan

**Affiliations:** ^1^Department of Nutrition and Food Science, University of Maryland, College Park, MD, United States; ^2^University of Maryland Extension, College of Agriculture and Natural Resources, College Park, MD, United States; ^3^Center for Food Safety and Security Systems, University of Maryland, College Park, MD, United States

**Keywords:** food waste, food spoilage, microbial (cross)contamination, quantitative microbial spoilage risk assessment, smart packaging technology, food date labeling

## Abstract

Ensuring a safe and adequate food supply is a cornerstone of human health and food security. However, a significant portion of the food produced for human consumption is wasted annually on a global scale. Reducing harvest and postharvest food waste, waste during food processing, as well as food waste at the consumer level, have been key objectives of improving and maintaining sustainability. These issues can range from damage during processing, handling, and transport, to the use of inappropriate or outdated systems, and storage and packaging-related issues. Microbial growth and (cross)contamination during harvest, processing, and packaging, which causes spoilage and safety issues in both fresh and packaged foods, is an overarching issue contributing to food waste. Microbial causes of food spoilage are typically bacterial or fungal in nature and can impact fresh, processed, and packaged foods. Moreover, spoilage can be influenced by the intrinsic factors of the food (water activity, pH), initial load of the microorganism and its interaction with the surrounding microflora, and external factors such as temperature abuse and food acidity, among others. Considering this multifaceted nature of the food system and the factors driving microbial spoilage, there is an immediate need for the use of novel approaches to predict and potentially prevent the occurrence of such spoilage to minimize food waste at the harvest, post-harvest, processing, and consumer levels. Quantitative microbial spoilage risk assessment (QMSRA) is a predictive framework that analyzes information on microbial behavior under the various conditions encountered within the food ecosystem, while employing a probabilistic approach to account for uncertainty and variability. Widespread adoption of the QMSRA approach could help in predicting and preventing the occurrence of spoilage along the food chain. Alternatively, the use of advanced packaging technologies would serve as a direct prevention strategy, potentially minimizing (cross)contamination and assuring the safe handling of foods, in order to reduce food waste at the post-harvest and retail stages. Finally, increasing transparency and consumer knowledge regarding food date labels, which typically are indicators of food quality rather than food safety, could also contribute to reduced food waste at the consumer level. The objective of this review is to highlight the impact of microbial spoilage and (cross)contamination events on food loss and waste. The review also discusses some novel methods to mitigate food spoilage and food loss and waste, and ensure the quality and safety of our food supply.

## 1. Introduction

Food loss and waste are major issues impacting food security and sustainability. Food waste can be broadly defined as any food item destined for human consumption that is removed from the manufacturing, retail, or consumer stages for a myriad of reasons, resulting in waste. Specifically, the United Nations World Food Program (UN WFP) defines food waste as “the discarding of food still fit for human consumption, either before or after spoilage occurs, at the retail, food service, or consumer levels.” In essence, food waste refers to food being discarded or sent to the landfill at the retail, food service provider, or consumer level. On the other hand, food loss describes the decrease in quality or quantity of food, making it unfit for human consumption, resulting from actions by food suppliers in the food supply chain, excluding retailers, food service providers, and consumers [World Food Program USA (WFP USA), 2022]. According to the United Nations Environment Program’s Food Waste Index Report of 2021, approximately 931 million tons of food waste was generated in 2019 at the consumer, retailer, and food service stages [United Nations Environment Program (UNEP), 2021]. Based on available data, this accounted for an estimated 17% of all food produced in 2019 being discarded at the supply chain, retail, or consumer level. Reducing food waste from a food security perspective is extremely important, especially in today’s world where an increasing proportion of the world population is suffering from hunger and malnutrition [United Nations (UN), 2023a]. From the sustainability viewpoint, food waste can be seen as a cumulative loss in (or wastage of) all resources, such as land, water, labor, money, and energy, used in its production [United Nations (UN), 2023a]. Therefore, reducing food loss and waste, in the form of “Responsible Consumption and Production” is one of the Sustainable Development Goals set by United Nations to achieve a more sustainable world by 2030 [United Nations (UN), 2023b].

An estimated 31% of all food produced for human consumption in the U.S. is wasted annually at a consumer and post-harvest level. Meat, poultry, and fish; vegetables; and dairy products (30, 19, and 17%, respectively) are believed to be the top three food groups in terms of the total value of loss (Buzby et al., 2014). Food loss and waste at the food supply chain, retail, and consumer levels can be attributed to a number of causes – damage due to unfavorable or extreme weather conditions, damage caused by insect, microbial, and vector activity, overproduction to account for uncertainty in consumer needs/expectations, non-conformance with industry or government food safety regulations, presence of cosmetic defects that would make the food undesirable to consumers, equipment malfunction, human handling resulting in damage to packaging material, lack of consumer demand for an overproduced food, spillages, over-zealous removal of edible parts during preparation, and lack of knowledge about the “best before” and “use by” date labels are a few of the most prominent causes of food loss listed by Buzby et al. (2014) in their report on food loss and waste in the U.S.

It is important to note that, although a large portion of food loss and waste (~61%) in developed countries such as the U.S. is attributed to waste at the consumer level, approximately 40% is due to loss at the supply chain level, including production, processing, storage, and transportation [United Nations Environment Program (UNEP), 2021]. While a number of studies, surveys, and reviews have postulated on the most common causes of food being lost in the food supply chain (which, for the purposes of this review, is defined as all stages of food production and preparation from growing and harvesting up to the retail stage) the exact proportion being attributed to each cause remains unknown. Overall, food waste can comprise both the edible and inedible portions of the food, such as the peels or inedible seeds of fruits and vegetables, the husk of cereal grains, or the sinews, fat, and carcasses of food animals. However, since inedible waste does not fit the scope of this review, we only focus on edible food waste (henceforth referred to as ‘food waste’).

Food spoilage is broadly described as any change in appearance, flavor, odor, microbial composition, or nutritional value that would impact the food product in such a way as to deem it unacceptable for human consumption (Rawat, 2015). Food is composed of a wide variety of nutrients, which, combined with a favorable environment, provides a conducive environment for the growth of relevant, site-specific spoilage and pathogenic microorganisms. This knowledge forms the basis for the current regulatory framework on the management and storage of foods in the food supply chain, as well as the guidelines for safe food management provided to consumers. Thus, it can be argued that microbial activity and spoilage are significant contributors to food waste generation, especially since spoiled food typically cannot be re-used or re-purposed. However, despite extensive analyses and reports on estimates of food loss and waste at each level along the supply chain and beyond, the proportion of food being lost or wasted due to microbial spoilage alone remains unknown. Furthermore, the impact of consumer knowledge regarding date labels on food items, specifically with regards to spoilage-related food waste, must be re-evaluated in the light of emerging ‘smart’ technologies.

This review aims to provide a review of the role played by food spoilage and spoilage-related activities in the generation of food waste. With this aim, we identify the chief causes of spoilage across the food supply chain (pre-retail, retail, and consumer levels). We additionally identify currently available and novel technologies and techniques, such as smart packaging, quantitative microbial spoilage risk assessments (QMSRAs), edible anti-decay peels, and Internet of Things (IoT) that could be implemented to reduce food waste, particularly from the perspective of minimizing food spoilage at both the production and processing and retail and consumer levels. We also discuss bringing food date labeling to the 21^st^ century (using smart labeling) to help minimize consumer uncertainty regarding food spoilage, and highlight the importance of education and social interventions in minimizing food spoilage and waste.

## 2. Major spoilage microorganisms in perishable foods

Spoilage microorganisms grow in all classes of foods – produce; grains; meats, poultry, and fish; animal products, such as dairy and eggs; and processed foods. The type of microorganism that impacts each class of food may differ, depending on the intrinsic properties of the food itself, as well as the external environment in which the food is being held. In this section, we focus on some of the major spoilage microorganisms in meat and meat products, seafood, fresh and fresh-cut produce, milk and dairy products, and eggs (Table 1). Table 2 also lists some major signs of spoilage in these food groups.

**Table 1:** Major signs of spoilage in various food groups (Silliker, 1980; Roberts et al., 2005).

Food group	Processing type		Signs of spoilage	Microbial agents of concern
Meat	Frozen		Visible mold colonies (1–4 mm) of black or white spot	*Cladosporium herbarum*, *Penicillium hirsutum*, *Cryptococcus*, *Trichosporon*, *Candida*
	Raw, comminuted		Souring	Gram-positive bacteria - *B. thermosphacta* and lactic acid bacteria
	Dried		Changes in appearance	*Aspergillus glaucus*
	Raw, cured		Sour or putrid odors, pin-holes, and gas pockets	Enterobacteriaceae, *Clostridium*, and lactic acid bacteria
	Cooked, uncured		Offensive and sickly odors; gas formation	Gram-negative psychrotrophic bacteria – *Pseudomonadaceae* and *Enterobacteriaceae*; lactic acid bacteria and *Brochothrix thermosphacta*; *Clostridium*; *Mucor*, *Penicillium, Rhizopus,* and *Aspergillus* spp.
	Cooked, cured		Souring, gas formation, greening, and surface softening	Heat resistant psychrotrophs, *Bacillus* spp., lactic acid bacteria, and *Brochothrix thermosphacta*
Poultry	Frozen raw		Black spots, whisker-like growth, white spots	*Cladosporium herbarum, Thamnidium elegans*, and *Sporotrichum carnis*
	Perishable, cooked		Strong offensive odors, small holes, pink discoloration, a milky exudate, gassiness, and sourness	Lactic acid bacteria, *Aeromonas* spp., and psychrotrophic clostridia
	Chilled raw		Off-odor progressing to slime formation	Psychrotrophic bacteria
	Heat-processed *OR* cured		Same as above, depending on post-processing storage temperature and time	Psychrotrophic bacteria *OR* molds, respectively
	Irradiated		Changes in color, odor, flavor	*Moraxella,* Enterococci, lactic acid bacteria
Seafood	Aquaculture		Off odors	*Pseudomonas* and *Acinetobacter*
	Frozen or chilled		Production of volatile amines	*Pseudomonas* and *Acinetobacter–Moraxella* spp.,
	Pasteurized		Putrid offensive off odors, rancidity, and textural changes	Spore forming gram-positive bacteria, and clostridia
	Freshly caught, iced >14 days		Dulled eyes, faded gills, slimy skin, offensive odor	Gram-positive Coryneforms, *Micrococcus*, *Bacillus*, *Staphylococcus*, *Pseudomonas*, and *Alteromonas*
	Processed – cured, canned		NA	*Clostridium botulinum*, yeasts
Vegetables	Raw, minimally processed		Soft rots and other rots, spots, blights, and wilts	Coliforms, *Erwinia Carotovora*, *Pseudomonas* spp., and *Clostridium* spp.
	Frozen		NA	Lactic acid bacteria, *Leuconostoc mesenteroides*, enterococci, micrococci and gram-positive and gram-negative rods
	Canned		Off-odors and swelling of the can	*Clostridium botulinum*
	Fermented, acidified		Pink color, softening of the flesh	Yeasts, lactobacilli, and *Bacillus* spp.
	Sprouts		Wilts rapidly, turns brown, flavor changes, and slimy decay	*Klebsiella pneumoniae* and *Enterobacter aerogenes*
	Mushrooms		Malformed stipes, fruiting body, brown blotching of the pileus, watery stipe, and dieback disease	*Verticillium fungicola*, *Pseudomonas tolaasii*, *Pseudomonas fluorescens,* yeasts, and molds
Fruits			Various types of rots (blue rot, green rot, gray rot, black rot, etc.)	Fungal genera, such as *Penicillium*, *Sclerotinia*, *Aspergillus, Rhizopus*, and *Botrytis* spp.; *Saccharomyces*, *Hanseniaspora*, *Pichia*, *Kloeckera*, *Candida*, and *Rhodotorula*; *Alternaria citri*, *Aspergillus niger*, and *Alternaria alternata*
Milk and other dairy products	Raw milks		Malty, rancid, yeasty, bitter, fruity, putrid off flavors, appearance of purple and reddish pigments, and ropiness	*Streptococcus lactis*, *Alcaligenes viscosus*, *Flavobacterium*, *Pseudomonas*, *Micrococcus*, *Bacillus*, *Enterobacter*, *Aeromonas*, *Chromobacterium* spp. and *Serratia* spp.
	Processed (or pasteurized) milks		Putrid, unclean, and fruity off-flavors, fat coagulation, bitterness, and ropiness	Gram-negative bacterium, such as *Pseudomonas*, *Flavobacterium*, *Chromobacterium*, *Alcaligenes*; pasteurization-resistant spore-formers such as *Bacillus* spp.
	Cream		Sweet curdling and bitter cream	*Bacillus* spp., *Pseudomonas* spp., and yeasts
	Concentrated milks		Off-flavors and blowing of cans	Xerophilic yeasts, *Bacillus stearothermophilus*, *Bacillus coagulans*, and *Bacillus licheniformis*
	Ice cream and frozen dairy products		Curdling and proteolysis	*Bacillus cereus*
	Fermented milks		Blowing (production of CO_2_), off-flavors and off odors, while mold growth is usually visual	Acid-tolerant fungi
	Cheese	Fresh cheese/ After few days of ripening	Early blowing	Coliforms and *Bacillus subtilis*
		Cured cheese (storage or ripening)	Late blowing (due the formation of butyric acid leading to the formation of gas and off-flavors)	*Clostridium tyrobutyricum* and *Clostridium butyricum*
			Mold formation, musty off-taints and odors, gas formation, off-odors, and visual defects	*Penicillium*, *Mucor*, *Monilia*, *Aspergillus*, *Cladosporium*, etc
Eggs	Shell eggs		Whiskers	*Cladosporium herbarum*
			Green	*Pseudomonas*
			Colorless	*Acinetobacter, Moraxella*
			Red	*Pseudomonas, Serratia*
			Fluorescence in the white	*Pseudomonas putida*
			Pink tinge in the white	*Pseudomonas fluorescens*
			Nutty odor, streaks of ferric sulfide on the surface	*Pseudomonas maltophilia*
			Blackening of the yolk	*Proteus vulgaris, Aeromonas*, and *Alcaligenes faecalis*
	Liquid eggs		Off odors and coagulation	*Alcaligenes*, *Proteus*, and *Flavobacterium*
Cereals and cereal products	Flours, starches, and meals		An odor of acetic acid and esters, acid fermentation, alcoholic fermentation	Lactic acid bacteria, *Acetobacter*, and *Bacillus* spp.
	Dough		Undesirable odor and flavor	*Leuconostoc mesenteroides*, and *Lactobacillus* spp.
	Breads		Visible fungal growth, rope, formation of red color	*Penicillium* spp. (especially *Penicillium roqueforti*), *Aspergillus* spp., *Bacillus subtilis*, and *Serratia marcescens*
	Pasta and noodles		Gas production	*Enterobacter (Aerobacter) cloacae*
Nuts, oilseeds, and dried legumes	Oilseed		Hydrolytic or “soapy” rancidity	Molds
Cocoa, chocolate, and confectionery	Chocolate		Soapiness	*Bacillus* spp. and molds
	Cocoa powder		Off-flavors	Molds
	Confectionery products		Gas formation causing fractures or bursting of products, slime formation, color changes or off-odors and off-flavors,	Xerophilic yeasts and molds, and *Zygosaccharomyces rouxii*

**Table 2:** Major spoilage and pathogenic microorganisms in various food groups.

Food group	Subtype	Predominant features	Major spoilage microorganisms	Major pathogenic microorganisms	Reference
Meat and meat products	Poultry	Bacteria: off-odors, and surface slime	*Acinetobacter* spp., *Pseudomonas* spp., and *Shewanella putrefaciens*.	*Campylobacter* spp., *Corynebacterium* spp., and *Listeria* spp.	Cerveny et al. (2009), Jay et al. (2005), Morales et al. (2016), Russell et al. (1995)
	Red meat	Bacteria: off-odors, off-flavors, discoloration, and gas production Yeasts and molds: whiskers, black spot, white spot, slime, and sticky surfaces	*Acinetobacter* spp., *Pseudomonas* spp., and lactic acid bacteria; *Cladosporium herbarum*, *Chrysosporium pannorum, Mucor racemosus*, *Rhizopus* spp., *Torulopsis* spp., and *Thamnidium elegans*.	*Aeromonas* spp., and *Enterobacteriaceae* spp.	Alía et al. (2016), Lozano-Ojalvo et al. (2015), Lund et al. (2000), Mills et al. (2014), Sun and Holley (2012)
	Processed meat and poultry	Bacteria: off-odors, and slime	*Pantoea* spp., and *Pseudomonas* spp.	*Streptococcus* spp. and *Salmonella* spp.	Jay et al. (2003), Kim et al. (2021), Sharma et al. (2013)
Seafood	Not available/Not applicable (NA)	Bacteria: distinctive off-odor	*Aeromonas* spp., *Brochothrix* spp., *Carnobacterium* spp., *Photobacterium* spp., *Pseudomonas* spp. (*P. fragi*, *P. putida*, *P. fluorescens*, and *P. vranovensis*), *Psychrobacter* spp., and *Shewanella* spp. (*Shewanella algae* and *Shewanella baltica*).	NA	Gennari et al. (1992), Vogel et al. (2005), Françoise (2010), Laursen et al. (2006), Macé et al. (2012), Møretrø et al. (2016), Hoel et al. (2019)
Fresh and Fresh-cut produce	Vegetables	Bacteria: bacteria soft rot including mushy texture, watery appearance, and lesions Yeats and molds: gray rot, *Rhizopus* soft rot, and sour rot	*Erwinia carotovora* (primarily at temperature between 30 and 35°C), and fluoresecent *Pseudomonas* spp. (primarily at refrigeration temperatures); *Botrytis cinerea*, *Geotrichum candidum*, and *Rhizopus stolonifera*.	*Clostridium botulinum*, *Corynebacterium* spp., and *Listeria monocytogenes*.	Bhat et al. (2010), Liao (2006), Liao and Wells (1987), Lilly et al. (1996), Olsen et al. (2006), Robbs et al. (1996), Ryser and Marth (2007), Shelake et al. (2022), Tournas (2005), Uzeh et al. (2009)
	Fruits	Bacteria: *Erwinia* soft rot Yeasts and molds: *Rhizopus* soft rot, gray rot, blue mold rot, and green mold rot	*Erwinia carotovora* and *Pseudomonas* spp. (mildly acidic to neutral pH); *B. cinerea*, *Penicillium* spp. (*P. expansum*, *P. digitatum*, and *P. italicum*), and *R. stolonifer* (pH < 4.0).	NA	Baggio et al. (2016), Bruton et al. (1991), Elmer and Reglinski (2006), Spotts et al. (1999), Ukuku et al. (2006)
Milk and other dairy products	Raw milk	Bacteria: unacceptable off-odors and flavors, increased viscosity, and rancidity caused by lipolytic and proteolytic activity Yeasts and molds: unacceptable off-odors and flavors	*Acinetobacter* spp., gram-positive lactic acid bacteria such as *Lactococcus* spp., and *Leuconostoc* spp., *Micrococcus* spp., and *Pseudomonas* spp.	*Bacillus* spp., *Enterobacteriaceae* such as *Hafnia alvei*, *Serratia marcescens* and *Citrobacter freundii, Microbacterium* spp., *Stenotrophomonas* spp., *Staphylococcus* spp., *Streptococcus* spp.; *Aspergillus glaucus*, *Aspergillus versicolor*, *Candida* spp. (*C. pseudoglaebosa*, *C. parapsilosis*, and *C. zeylanoides*), and *Scopulariopsis* spp.	Baur et al. (2015), Ercolini et al. (2009), Lima and Santos (2017), von Neubeck et al. (2015), Quigley et al. (2011)
	Pasteurized milk	Bacteria: undesirable off-odors and flavors, body defects, and pigment production	*Acinetobacter* spp., *Citrobacter* spp., *Flavobacterium* spp., *Paenibacillus* spp., and *Pseudomonas* spp., (*P. fluorescens, P. fragi,* and *P. lundensis*).	*Aeromonas* spp., *Bacillus cereus*, *Enterobacter* spp., and *Klebsiella* spp.	Martin et al. (2018)
	Cheese	Bacteria: early blowing, off-odor, off-flavor, body defects, pigment defects, and gas defects Yeasts and molds: mold formation	Lactic acid bacteria and *Pseudomonas* spp.; *Debaryomyces hansenii*, *Geotrichum candidum*, *Kluyveromyces marxianus*, *Kluyveromyces lactis*, *Rhodotorula mucilaginosa*, *Penicillium* spp., and *Phoma glomerata*.	*Clostridium* spp., *Enterobacter aerogenes*, *Escherichia coli*, and *Klebsiella aeogenes*.	Casalinuovo et al. (2015), Geronikou et al. (2020), Alichanidis (2007), Caldera et al. (2015), Le Bourhis et al. (2007), Ortakci et al. (2015)
	Yogurt and other dairy products	Yeasts and molds: Yeasty odors, bitter flavors, and gas production	*Clavispora lusinaniae*, *Penicillium* spp., and *Torulaspora delbrueckii*.	NA	Buehler et al. (2017)
Eggs	NA	Bacteria: green rot, colorless rot, pink rot, black rot, and characteristic sulfurous odor Yeasts and molds: whiskers and pinspots	*Acinetobacter* spp., and *Pseudomonas* spp.; *Cladosporium herbarum*, and *Penicillum* spp.	*Aeromonas* spp., *Enterobacter* spp., *Escherichia* spp., *Proteus* spp., and *Serratia* spp.	Fa Mansour et al. (2015), Geornaras and Sofos (2004), Jones and Musgrove (2008), Sauter et al. (1962)
Grains/cereals	NA	Bacteria: discoloration, loss of weight, and bad odor Yeasts and molds: discoloration, loss of weight, and bad odor	*Bacillus* spp.; *Cladosporium* spp., *Fusarium* spp., and *Penicillium* spp.	*Salmonella* spp.; *Aspergillus* spp., and *Eurotium* spp.	Magan and Aldred (2006)
Oils/oilseeds	NA	Yeasts and molds: soapy rancidity, bad odors, and flavors	*Aspergillus* spp., *Fusarium* spp., and *Penicillium* spp.	NA	Palumbo and Harris (2011)
Processed food	Breads	Bacteria: sticky and stringy degradation of the crumb, slime formation, discoloration, and an odor reminiscent of rotting fruit Yeasts and molds: development of white chalk like spots	*Bacillus subtiles*; *Penicillium* spp., and *Rhizopus stolonifer*	NA	Pacher et al. (2022), Singh and Anderson (2004)

### 2.1. Conditions conducive to spoilage microbial growth – an overview

Based on intrinsic bacterial characteristics and other external conditions, spoilage microbial organisms colonize and grow in specific food items at different stages in the food supply chain, which, in turn, leads to food loss and waste. Parameters that can influence the growth of spoilage microorganisms can be divided into (i) intrinsic and (ii) extrinsic parameters. (i) Intrinsic parameters are the physical and chemical properties of the food itself, and include water activity, pH, and availability of nutrients. (ii) Extrinsic parameters include environmental factors where food is stored, such as the temperature, availability of oxygen, and humidity (Veld, 1996; Sperber, 2009; Mena et al., 2014).

### 2.2. Meat and meat products

Sufficient nutrient composition, appropriate pH, and high water activity of meat and meat products make microbial spoilage very common in the meat industry.

Poultry spoilage is majorly driven by bacteria, though molds and yeasts also play active roles in the spoilage. *Pseudomonas* spp., including both fluorescent and non-fluorescent strains, are the prevalent spoilage bacteria in poultry (Morales et al., 2016). Surprisingly, *Shewanella putrefaciens*, which is commonly considered a marine bacterium, is a prevalent cause of poultry spoilage (Russell et al., 1995). Undesirable off-odors caused by amino acid metabolism, and the subsequent development of surface slime are the predominant features of poultry spoilage (Cerveny et al., 2009).

The spoilage of red meat is determined by its surface conditions (Wickramasinghe et al., 2019). The most common bacterial species responsible for red meat spoilage are *Acinetobacter* spp., *Pseudomonas* spp., and lactic acid bacteria (Mills et al., 2014). The key spoilage characteristics of red meat are off-odors, off-flavors, discoloration, and gas production (Sun and Holley, 2012). Dried meat surfaces favor the growth of psychrotrophic molds and yeast such as *Rhizopus* spp. and *Torulopsis* spp. (Mendonca, 2010). Major fungal spoilage-related defects in red meats are whiskers, black spot, white spot, the development of slime, and sticky surfaces (Alía et al., 2016).

Bacterial species implicated in processed meat and poultry spoilage are highly dependent on the presence of conducive conditions, such as nutrient (such as water) and oxygen availability. For example, spoilage in ground meat and poultry could be attributed to bacteria with varying oxygen requirements. For example, aerobic bacteria would thrive in ground meat and poultry due to the introduction of oxygen during the grinding and mixing process (Jay et al., 2003; Maćkiw et al., 2011; Rooney et al., 2011; Sharma et al., 2013).

### 2.3. Seafood

Seafood is one of the most perishable foods due to its high water content, neutral pH, and adequate nutrients. Spoilage in seafood can be distinguished by a distinctive, unacceptable off-odor. *Shewanella* spp. and *Pseudomonas* spp., which could break down proteins in seafood and produce off-odors, are two bacterial species that prominently feature in the microbial spoilage of seafood (Wang et al., 2017, 2021). The specific species that could be responsible for spoilage varies according to extrinsic factors such as the temperature (Parlapani and Boziaris, 2016). A number of other bacteria, such as *Carnobacterium* spp. and *Brochothrix* spp. are also actively involved in the spoilage of seafood (Gennari et al., 1992; Laursen et al., 2006; Møretrø et al., 2016; Hoel et al., 2019).

### 2.4. Fresh and fresh-cut produce

Fresh and fresh-cut produce, which undergo minimal processing (with minimal alterations to their ‘fresh’ nature), could naturally carry microorganisms and thereby increase the probability of microbial spoilage (Harris et al., 2003).

The leading bacterial spoilage microorganism related to spoilage of fresh and fresh-cut vegetables is bacterial soft rot, caused by *Erwinia carotovora* and fluoresecent *Pseudomonas* spp. (Liao and Wells, 1987; Olsen et al., 2006; Shelake et al., 2022). Among the fungal sources of fresh and fresh-cut vegetable spoilage are gray rot caused by *Botrytis cinerea* (Romanazzi and Feliziani, 2014), *Rhizopus* soft rot caused by *Rhizopus stolonifera* (Singh and Afaque, 2021), and sour rot caused by *Geotrichum candidum* (Tournas, 2005).

The spoilage microbiota in fresh and fresh-cut fruits is largely dependent on the pH of the fruit. Melons, with a mildly acidic to neutral pH, are primarily impacted by *E. carotovora* and *Pseudomonas* spp., which also affect fresh and fresh-cut vegetables, depending on the storage temperature (Bruton et al., 1991; Ukuku et al., 2006). Fruits with low pH (<4.0) and relatively high concentrations of sugar like tree fruits, citrus fruits, and berries are particularly susceptible to spoilage by molds and yeast, while the growth of most bacteria is inhibited (Rawat, 2015). In fresh-cut fruits, on the other hand, yeast has been known to convert sugars in the fruit to CO_2_ and ethanol, leading to spoilage (Barth et al., 2009). Molds such as *R. stolonifer, B. cinerea,* and *Penicillium* spp. such as *P. expansum, P. digitatum, and P. italicum* are capable of causing a broad range of soft-rot spoilage in fruits including *Rhizopus* soft rot, gray rot, blue mold rot, and green mold rot (Spotts et al., 1999; Elmer and Reglinski, 2006; Baggio et al., 2016).

### 2.5. Milk and dairy products

Milk and dairy products are rich in water, fats, proteins, and vitamins that support the growth of diverse groups of microorganisms. Psychrotrophic bacteria, which can survive at low temperatures, are predominantly involved in the spoilage of milk and dairy products, which are commonly stored under refrigerated conditions.

Raw milk, which contains a near-complete nutritional profile and has a neutral pH, is an optimal place for the growth of a large variety of microorganisms. Gram negative bacteria make up a majority of the bacterial species contaminating raw milk. On the other hand, high amounts of gram-positive bacteria cause sourness in raw milk (Quigley et al., 2011). Molds and yeasts are capable of spoiling raw milk as well (Baur et al., 2015).

Pasteurized milk spoilage is typically caused by heat-stable bacteria such as *Citrobacter* spp. and the heat-stable lipolytic and proteolytic enzymes produced by *Pseudomonas* spp. (von Neubeck et al., 2015; Martin et al., 2018; Center for Dairy Research (CDR), 2020). These bacteria are characterized by the ability to survive under low temperatures, heat resistance capacity in proportion to their normal growth temperature, and the capacity to produce heat-stable extracellular enzymes such as proteases and lipases, which could result in undesirable off-odors and flavors (Reichler et al., 2019).

Cheese spoilage is typically caused by fungi, which can survive under low pH and low a_w_. Cheese products are also susceptible to both gram-negative and gram-positive bacteria, which are responsible for early blowing (Alichanidis, 2007), off-odor, off-flavor, body defects (such as dryness, too much moisture, or mechanical holes), and pigment defects (Caldera et al., 2015) and gas defects in cheese (Le Bourhis et al., 2007; Ortakci et al., 2015).

Yogurt and other dairy products are susceptible to spoilage by a broad range of yeasts and molds. Yeasts are the most prevalent spoilage organism in yogurt and other dairy products. Yeasty odors, bitter flavors, and gas production are the key characteristics of fungal spoilage in yogurt and other dairy products (Ledenbach and Marshall, 2009).

### 2.6. Eggs

Eggs are susceptible to spoilage by gram-negative bacteria and filamentous molds, even though they possess a system of barriers such as the shell, and the antimicrobials and high alkaline pH (7.6–9.2) of egg whites (Stadelman and Cotterill, 1995). Bacterial spoilage of eggs is characterized by “rots” (Jones and Musgrove, 2008; Fa Mansour et al., 2015). Another typical feature of bacterial spoilage of eggs is the characteristic sulfurous odor, associated with the metabolism of amino acids, which produce H_2_S and odorous compounds (Jan et al., 2018). “Whiskers” and “pinspots” are the common features of mold spoilage on the surface and internal portions of eggs, respectively (Geornaras and Sofos, 2004).

## 3. Routes of microbial food spoilage and loss/waste in the food supply chain

**Figure 1:**
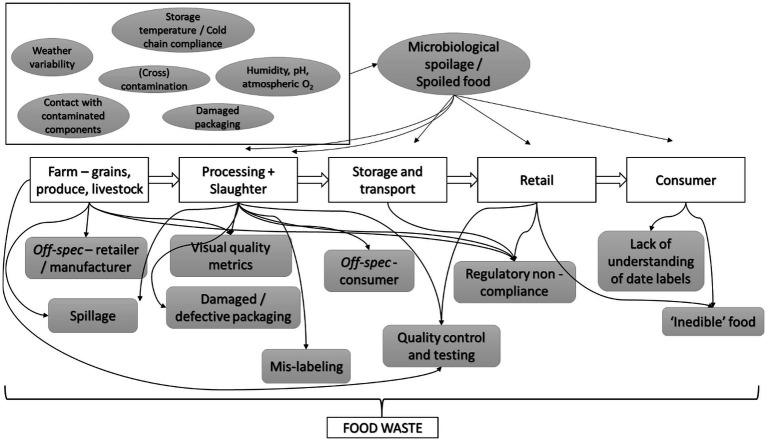
Food waste causes, including factors contributing to microbiological spoilage, across the food supply chain, at retail, and the consumer stage.

Food passes through several stages along the supply chain – farm or raw materials stage, processing, storage, transport, and retail – before reaching the consumer. Food loss and waste occurs at all stages along this chain; however, the root cause behind the generation of loss or waste does not necessarily occur at the stage where the food is discarded (Papargyropoulou et al., 2014; Göbel et al., 2015). Food undergoes a certain amount of *scarring* from the processes that are meant to enhance its quality, safety, and longevity. From a microbiological perspective, this, in turn, could result in microorganisms migrating to, and contaminating, the otherwise sterile interior portion of some foods such as eggs (Sperber, 2009). On the other hand, a large portion of food is discarded during production, processing, and retail, due to the product being *off-spec* or not adhering to consumer likes or regulatory standards. In this section, we identify the major causes of food loss and waste at the individual modules of the food supply chain up to and including retail, with a focus on the causes of microbial spoilage (Figure 1). It must be noted that, since this section focuses primarily on food loss and waste due to microbial (cross)contamination or spoilage, it does not cover intentional causes of food loss or waste, such as destructive testing for quality control and assessment, or losses caused during product formulation or parameter adjustment (especially in the case of prepared, ready-to-cook, or ready-to-eat foods), which form a significant portion of food loss and waste, which have been covered in greater detail elsewhere (Raak et al., 2017) and have been summarized in Table 3.

**Table 3:** Causes of food waste across the food supply chain and in the retail and consumer levels.

Harvest/Farm stage	Processing/slaughter	Packaging, storage, transport	Retail	Consumer
Mechanical damage during harvest operation	Contamination	Poor temperature control	Poor temperature control	Improper storage
Spillage	Damage during transit/transport	(Cross)contamination	Improper handling by retail staff, consumers	Perception of quality based on date label
Sorting	Removal of unpalatable portions (cutting, peeling, etc.)	Damage during packaging	Discarding of entire package when one item is visibly spoilt/contaminated	Purchasing more than required
Grading of food – regulatory non-compliance	Shelf-life management	Damage during storage	Demand variability – low sales	Removal of perceived inedible parts
Non-compliance with supplier/retailer/consumer needs	Poor quality raw material (spoiled vegetable, unhealthy animal, etc.)	Delays during shipping	Inventory management	Microbiological spoilage
Insect and pest predation	Over-trimming during cutting	Weather variability	(Cross)contamination	Temperature abuse
Microbiological spoilage	Regulatory non-compliance	Variability in sales	Quality control	
Storage issues	Weather variability	Improper labeling	Weather variability	
Weather variability	Storage issues	Using incorrect packaging (re-bagging)		
Product deterioration due to prolonged storage/transport	Spillage	Damage from mishandling of packaged goods		
Destructive quality control and testing	Destructive quality control and testing	Damage during transport (incorrect stacking, etc.)		
Preparing for predicted sales	Preparing for predicted sales	Insufficient sanitation		
Overproduction to meet expected/forecasted demands	Technical/equipment issues/malfunction			
	Blackouts/electrical issues			
	Insufficient sanitation			

### 3.1. Farm

Soil and water are primary sources of contamination in the pre-harvest, or farm, stages of the food supply chain. Soil, which houses both microorganisms such as bacteria, yeast, and mold, and vectors that carry these agents, is a direct contaminant for many food groups, including produce, cereals, and animal meat. Moreover, soil contains animal and bird feces, which act as reservoirs of microorganisms. Water acts as a vector for a number of bacteria, protozoans and parasites, and can contaminate foods during the harvest and pre-harvest stages (*via* irrigation; Ijabadeniyi et al., 2011). Additionally, wind carries mold, which causes mold spoilage of food. In animal and poultry operations, animal skin and animal feed, which also house many microorganisms, also act contaminating agents, especially when multiple animals are housed in close quarters (Sperber, 2009). Exposure of foods during these early stages, combined with environmental conditions that favor microbial growth (such as temperature), results in food spoilage or disease in animals/poultry. Eventually, spoiled food/diseased animals/birds would be discarded during the pre-harvest and harvest stages in order to protect and preserve others within the batch, pack, or flock.

### 3.2. Processing

The outer skin of fruits, vegetables, cereal grains, nuts and other plant-based foods, unless perforated in some way, is designed to keep out external contaminants, including those of microbial origin. Similarly, the skin and hide of meat animals are meant to protect the animal from microorganisms. Moreover, despite the presence of microorganisms in the intestinal tract of animals, the latter is essentially separated from the muscles and living tissues of the animal, leaving these parts sterile. However, processing tends to upset this fragile balance. Examples of processes that result in the sterility of the interior being compromised include removal of the bran from cereal grains, vigorous washing (particularly with contaminated water), chopping or juicing of fruits and vegetables, and removal of the hide and intestines (perforation of the intestine during this process could lead to very high contamination levels) and cutting and/or grinding of meat (which exposes a greater surface area of meat to microorganisms) (Sperber, 2009). These processes also expose food preparation, or food surface, areas to microorganisms, resulting in contamination and (cross)contamination of previously sterile food. Prior studies have reviewed the major causes of food loss in the processing module (Mena et al., 2014; Raak et al., 2017). Primarily, weather variations, poor post-harvest management, non-conformance with retailer or regulatory specifications, excessive waste during cutting or trimming, incorrect visual quality metrics (shape, size, etc.), and contamination or rot formation in the product were identified as causes of food being discarded. It is important to note that, although the exact proportion attributable to spoilage and contamination cannot yet be determined, deterioration in product quality due to (cross)contamination (of meat) and development of rots (in produce) remains a consistent factor contributing to food waste in the processing stages (Mena et al., 2014).

### 3.3. Handling

Human handling remains a chief cause of (cross)contamination in food processing facilities. The use of unclean hands or gloves, human physiological activities such as talking, coughing, or sneezing, movement between areas within the processing facility with different controls for microbial growth, as well as ineffective cleaning of food-handling utensils and equipment are some of the chief handling-related causes of microbial contamination, and eventually food waste (Sperber, 2009). An example of microbial (cross)contamination due to the use of unclean equipment is the outbreak of *Salmonella* from fresh-cut cantaloupes due to (cross)contamination with an unclean knife (Castillo et al., 2009). Water is another major point of contamination – the use of non-potable water for washing is a major cause of microbial spoilage, whereas improper storage of foods during cleaning and sanitation activities could result in direct contamination with aerosolized microorganisms from equipment nooks and crannies or the processing facility floors (Sperber, 2009). For example, the former was identified as the reason for a major *Salmonella* outbreak from fresh produce (oranges) in the U.S. (Parrish et al., 1997). This is also true for the spread and proliferation of microorganisms contributing to food spoilage across the food supply chain.

### 3.4. Transport and logistics

Food passing through the (required) transport and logistical stages in the supply chain is subject to mechanical damage, ranging from mild impacts and abrasions to severe structural damage. This, in turn, can promote microbial spoilage contamination and accelerate enzymatic degradation (Raak et al., 2017) in the damaged food item. Insufficient temperature management is another cause of microbial and physiological spoilage during transport. A high fraction of food loss and waste has been previously attributed to insufficient cold chain management – particularly during the summer months, when loss and wastage of fresh produce and meats tends to peak (Mena et al., 2014; Jedermann et al., 2014a; Hammond et al., 2015). However, the temperature at which foods, particularly fresh foods, are to be stored is also product-specific – for example, fresh tropical fruits would be susceptible to chilling injury and loss if transported under refrigeration temperatures (Jedermann et al., 2014c).

### 3.5. Packaging and storage

Food packaging acts as a primary barrier, protecting food from physical, chemical, and microbial contaminants. However, packaged foods are also susceptible to damage and spoilage from mishandling (causing physical defects), the use of already contaminated packaging material, or contact with a non-sterile environment (which can contain aerosolized microorganisms or spores) immediately prior to packaging. For example, a 1999 outbreak of *Salmonella* in the U.S. was attributed to unhygienic conditions where orange juice was mixed and bottled (Vojdani et al., 2008). Once packaged, food must be stored at the correct storage temperature – maintenance of a cold-chain across all stages of the food supply chain is extremely important, as described in section 3.4. It is also important to maintain and ensure cleanliness in all storage areas, particularly areas that are in direct contact with food, across the food supply chain. This is because unclean surfaces and areas can become significant contributors to cross-contamination, resulting in massive loss of food. For example, an inadvertent source of contamination during storage may be condensate formed in refrigeration units, which can carry microorganisms, and can be spread by the ventilation systems throughout the food-processing plants.

### 3.6. Retail

According to a survey of retail food service providers conducted by Teller et al. (2018) food waste at the retail level can be primarily attributed to lack of consumer demand, increased ordering of products, particularly seasonal products (related to forecasting of demand), and consumer intolerance to products that are slightly deformed or ‘ugly.’ The authors also attributed food waste to the range of brands available for a single product and the product(s) being too close to expiring at the time that they are delivered to the store. This was also validated by a descriptive analysis of the causes of food waste conducted by de Moraes et al. (2020). These authors also identified inappropriate storage, issues with transportation, and lack of stringent quality standards as drivers of spoilage-related food waste, although the percentage of waste attributed to these causes was relatively low (at a cumulative ~10%).

## 4. Consumer-level food waste – the role of date labeling

Although food loss and waste can occur at all levels along the supply chain, waste at the consumer level is one of the chief causes of food waste, with approximately 31% of food waste in North America occurring at the retail or consumer level (Houghton, 2020). At the consumer level, food waste is attributed to multiple factors, including, but not limited to, inadequate or improper storage; spillage; excessive trimming, cutting or peeling of foods; lack of knowledge about preparation; excessive purchasing of foods, leading to food aging or spoilage; uneaten food; and insufficient knowledge about date labels (Buzby et al., 2014). Of these, food waste associated with the misunderstanding of date labels has attracted more attention over the past few years (Patra et al., 2022). In fact, studies have shown that most consumers, irrespective of their educational qualifications, are unable to interpret information provided on food date labels correctly and confuse date labels with food safety, resulting in unnecessary food waste (Patra et al., 2022).

There are over 50 types of food date labels in the U.S.^1^ such as ‘Best Before’ and ‘Use by’ (Kavanaugh and Quinlan, 2020). Most date labels are indicators of food quality or freshness, while typically not being indicative of the safety of food products from a microbiological perspective (Eičaitė et al., 2021). However, most consumers, while being heavily reliant on date labels to evaluate the safety of their food, have an improper or incomplete understanding of food date labels; as a result, consumers tend to throw away food once the date on the label has passed. In fact, a study focused on consumer preferences for suboptimal food pointed out that consumers were highly dependent on the packaging date labels to determine whether foods were safe to eat or not (Huang et al., 2020). For example, Jones et al. (2021) mentioned that cartons of eggs that exceeded the ‘Best by’ date were typically discarded since consumers mistook this date as an indicator of expiration and thought the eggs were no longer safe to eat or were already spoiled. In essence, consumers remain unwilling to buy foods past their “Best by” dates, despite these foods not having any food safety issues, resulting in food waste (Huang et al., 2020). In fact, it has been pointed out that 410,000 tons of food are tossed every year in the U.K. due to the expiration of date labels (‘best before’ dates) though they are still safe to eat, with an additional 220,000 tons being discarded even before passing of the ‘best before’ dates, which was linked to a lack of consumer knowledge about how to use the date label information (Hall-Phillips and Shah, 2017; Liegeard and Manning, 2020).

## 5. Current and novel methods to minimize spoilage and/or reduce food waste

### 5.1. Sensing technologies and smart packaging

Although all sectors of the food supply chain and at the retail, food service, and consumer levels are involved in the generation of food waste, the high percentage of consumer-related waste necessitates the development and implementation of novel technologies to identify and potentially reduce food waste. Food packaging, in addition to providing essential product information to consumers, remains an essential method to ensure food safety, reduce enzyme and microbial activity, minimize exposure with the atmosphere, and overall increase shelf life (Poyatos-Racionero et al., 2018). Active and intelligent packaging technologies, particularly, could have a major impact on reducing food waste.

According to the European Union regulation EC/450/2009, intelligent packaging materials are those that monitor and indicate the condition of packaged food or the environment surrounding the food (Poyatos-Racionero et al., 2018). Traditionally, these have relied on the use of chemical indicators or coatings to communicate the characteristics of the food item to the processor, retailer, or consumer. For example, the chemical indicator could interact with food components or the metabolites in the headspace of packaged foods, or even with the storage environment, generating a visible response, such as a change in indicator color, which would correspond with the state of the food product. Recently, however, there has been an impetus to use more advanced indicators in smart packaging, such as radio frequency identification technologies (RFID), time–temperature indicators (TTIs), freshness indicators, chromogenic sensors, and global positioning system (GPS), which are increasingly being used to ensure food quality, fast communication, and contribute to better transport modalities and up-to-date information concerning shelf life (Raak et al., 2017; Poyatos-Racionero et al., 2018). Although currently available intelligent packaging systems have been extensively reviewed elsewhere (Vanderroost et al., 2014; Zhang et al., 2016), this study provides a brief overview of the most promising technologies to detect spoilage or quality issues in food, and thereby reduce food waste.

RFID tags are typically used to track and identify products, allowing for improved product traceability (Lee and Rahman, 2014), better inventory management and streamlined supply chain processes. RFID tags are particularly promising, since they allow storage of diverse streams of information, such as product source, environmental parameters, and expiration date. Such information would help ensure compliance with government regulations pertaining to food traceability (such as the Food Safety Modernization Act’s rule 204), Hazard Analysis and Risk Based Prevention Controls (HARPC), and cold chain management in a number of highly perishable food classes including fresh fruits and vegetables, frozen fish, and soft cheeses, while simultaneously allowing timely and accurate exchange of important information among trading partners. Examples of U.S. companies that have successfully implemented RFID technology for improved traceability, better cold chain management, demand management, and faster identification of products developed from contaminated or spoiled source material include Mission Foods, LaClare Farms, Chipotle, and Ste-Lor Oaks beef (Attaran, 2012; Littman, 2022). However, widespread implementation of this useful technology in the food industry has been impeded by high capital costs, lack of in-house expert knowledge, concerns regarding data privacy, and uncertainty regarding usage standards and regulations, among other issues (Attaran, 2012).

TTIs are highly efficient, relatively easy-to-operate indicators that continuously monitor the temperature of foods, particularly for refrigerated and frozen foods. TTIs are currently the most common system to identify physical, chemical, enzymatic, and microbial changes at a commercial level, primarily because they provide visual cues identifying changes in quality or freshness (Poyatos-Racionero et al., 2018), especially with major companies like 3M (3M™ MonitorMark™), the Cole-Parmer Instrument Company (Traceable ONE™, Traceable Excursion-Trac™, among others), and DeltaTRAK (WarmMark®) investing in developing novel, easy-to-decipher TTIs for the food industry (MarketWatch, 2023). However, these also have the potential disadvantages of spontaneous activation of chemicals, leading to false positive results; enzymatic instability; and leakage of the chemicals into the food product (Lin et al., 2021; Ye et al., 2022). Moreover, such technologies would not be cheap, since many of the available TTIs are not reusable.

Freshness indicators are another smart technology that could help detect freshness and quality of foods. These indicators highlight chemical changes occurring in food products during storage, such as changes in the concentration of metabolites like glucose, organic acids, carbon dioxide, biogenic amines, volatile nitrogen compounds or sulfur derivatives, which are potential signs of microbial growth (Arvanitoyannis and Stratakos, 2012; Poyatos-Racionero et al., 2018). Although a considerable amount of research has been conducted into freshness indicators such as ToxinGuard and SensorQ, and their efficacy in minimizing spoilage-related waste, there are currently very few that are commercially available (such as RipeSense®). It is important to note that a vast majority of these sensing technologies have been developed for use in foods packaged with modified atmosphere packaging (MAP) technology, which in itself was developed to increase shelf life and minimize microbial activity in packaged foods. However, their successful use in commercial applications remains rare. This could potentially be addressed by long-term validation of their safety (specifically for indicators that employ chemical components), improved regulatory mechanisms to allow their use in commercial applications, and increased research into the use of cost-saving materials in the development of these sensors.

**Figure 2:**
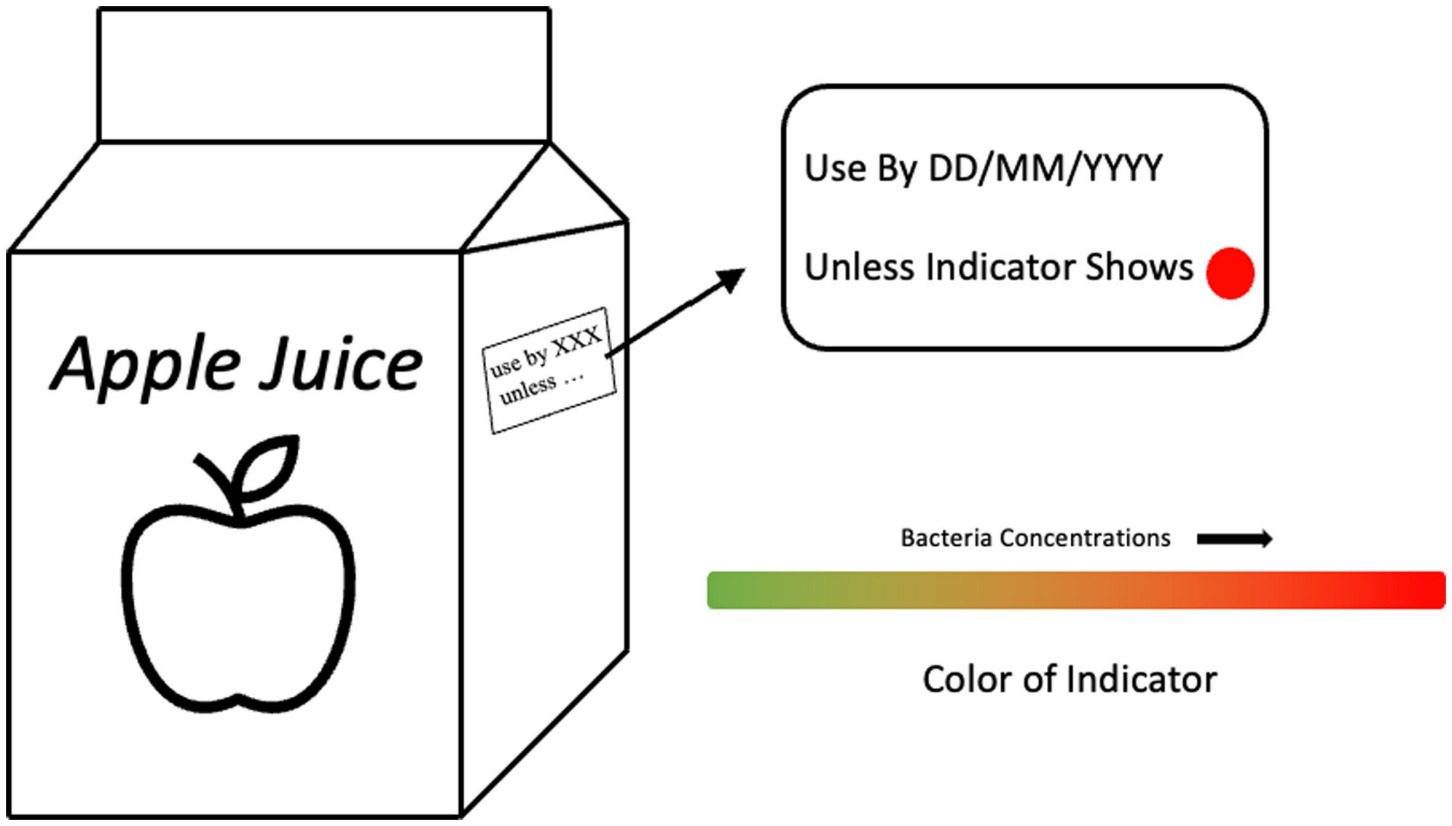
Suggested design for an intelligent date label. Here, we take the example of a time-temperature indicator (TTI) smart date label.

Recent studies have also proposed combining intelligent packaging techniques with food date labels (in a manner similar to the schematic shown in Figure 2) as an effective method to provide consumers with real-time information concerning the quality and safety of food products, thereby reducing food waste at the consumer level. Adopting TTI into food date labels enables the estimation of remaining shelf-life in a non-destructive manner. Labuza and Szybist (2008) state that instead of traditional open-date labels, food products could be labeled as “use by XXX unless indicator shows …,” based on TTI design specifications. TTI labels not only offer insights about the freshness of the food product to consumers by indicating the “Use By” date, but also guarantee the safety of food and reduce food waste as they convey the condition of food products on a real-time basis by adopting TTI. A great amount of gas-sensitive smart labels such as oxygen-, CO_2_-, and volatile compound-sensitive smart labels, have been reported to successfully convey up-to-the-minute information regarding the atmosphere in contact with food products (Saliu and Della Pergola, 2018; Wang et al., 2019; Niponsak et al., 2020). As such, gas-sensitive smart labels could be combined with conventional date labels (similar to TTI date labels) to help minimize food waste by supplying real-time data about the quality of food products. In addition, though not widespread, RFID smart labels have also demonstrated the potential to provide timely monitoring information for food products (Chen et al., 2023).

### 5.2. Quantitative microbial spoilage risk assessment

Although quantitative microbial risk assessment (QMRA) has been extensively applied to characterize the risk of foodborne illness, its application in food spoilage remains novel. However, over the past decade, studies have postulated on the importance of quantitative microbial spoilage risk assessment to develop more effective management strategies to minimize food spoilage, extend shelf life, and limit global food waste [European Food Safety Authority (EFSA), 2020]. Although the scope of any risk assessment would be to assess and manage the risk of the presence of a microbial agent in food, a QMSRA aims to assess both the growth of the spoilage microorganism of interest and its spoilage-associated metabolic activity. In essence, in addition to the growth response of the spoilage microorganism to prevalent environmental conditions such as the temperature, humidity, and oxygen content, this framework would have to incorporate models to predict the conditions that would lead to the development of off-flavors, odors, and textures, as well as the probability that a consumer would reject the food item based on their personal perception of spoilage (Koutsoumanis et al., 2021). Despite these differences, a few studies have developed comprehensive QMSRAs for various food-spoilage microorganism combinations. A majority of these risk assessments focus on quantifying spoilage risk from molds such as *A. fischeri* in pasteurized strawberry puree (Dos Santos et al., 2020), *A. niger* in yogurt (Gougouli and Koutsoumanis, 2017), and other molds in bread (Dagnas et al., 2017). Spoilage risk assessments have also been developed for bacterial spoilage agents such as *Geobacillus stearothermophilus* (canned green beans and bread; Rigaux et al., 2014; Pujol et al., 2015), as well as pathogenic agents responsible for food waste, such as *Clostridium botulinum* (ultra-high temperature pasteurized milk) and *Bacillus cereus* (bread; Pujol et al., 2015). However, these are few and far between, with a standardized framework for QMSRA being made available only as recently as 2021 (Koutsoumanis et al., 2021). The use of QMSRA would be particularly useful at the supply chain level, where the industry (producers and processors) can apply pre-set quality targets by developing and implementing effective control programs to minimize spoilage-related activities and thereby reduce food waste (Koutsoumanis et al., 2016). For example, QMSRA models could be used to simulate what-if scenarios with different combinations of product formulation, process and storage conditions, and packaging choices, which in turn could be used to assess the impact on spoilage microorganism growth and activity. If such models were to be adopted by food processors and retailers, they would help supplement the empirical decision making process currently being employed by managers in the food industry, particularly in selecting expiration and ‘best by’ dates that could minimize food waste at the retail and consumer levels (Koutsoumanis et al., 2021).

### 5.3. Other novel technologies – imaging, novel freshness peels, internet of things (IoT)

Other novel technologies that have recently been making waves in identifying or minimizing food spoilage include imaging technologies (such as hyperspectral imaging) to determine food freshness and quality in a non-invasive manner and biodegradable and edible food coatings such as Apeel that slow down the rate of water evaporation that causes fruits and vegetables to degrade while allowing for a natural exchange of gases with the atmosphere, which could be particularly useful in ensuring that produce reaches retail and the consumers before it spoils (Cosgrove, 2018; Milland and Taylor, 2018; Central Florida Store Services, 2020). Delaying produce ageing is another method being explored to extend produce shelf life and consequently minimize pre-retail food loss. The USDA-funded Hazel 100™ utilizes 1-methylcyclopropene (1-MCP) to reduce the production and absorption of ethylene, which is naturally produced by produce as it ages. This technology has proven to be effective in delaying ageing and reducing food loss in produce such as apples, cherries, limes, avocados, and melons (Fresh Fruit Portal, 2019; Diep, 2022). Hazel Technologies is also in the process of developing an antimicrobial-based technology (Hazel Endure™) to reduce mold spoilage in fruits such as grapes and berries (Hazel Technologies, 2023). In the meat industry, a novel nanofiber absorbent pad containing the antimicrobial polyhexamethylenebiguanide hydrochloride (PHMB) has been recently developed to reduce meat spoilage. This method combines antimicrobial technology with an anthocyanin-based indicator technology to easily detect meat spoilage. If this method proves to translate well into a commercial setting, it could have a significant impact on minimizing post-retail food waste (Jiao et al., 2023).

Another method that has seen growing interest by stakeholders in the food supply chain to reduce spoilage and food waste is IoT. Internet of Things or IoT is a platform allowing seamless communication between smart devices, enabling information sharing across platforms in a convenient manner (Vermesan et al., 2011; Marjani et al., 2017). In the context of the food supply chain, when a food product is assigned a digital footprint (such as one, or a series of, sensor(s), GPS technology, or cameras), the latter can be used to collect data on the food (humidity, oxygen content, quality metrics) and the surrounding environment (such as temperature and relative humidity) in the entire food supply chain (agricultural production, post-harvest handling, processing, transport, and storage) and beyond, as well as data on transport logistics and packaging. In this framework, information on the product can be collected using a range of sensors, such as temperature, humidity, chemical, and optical sensors. This data can then be processed for food quality monitoring using machine learning models such as clustering algorithms, decision tress, regression models, artificial neural networks, and support vector machine models (Ahmadzadeh et al., 2023). The results of these analyses can then be consolidated and utilized by the stakeholders to make critical decisions regarding strategies to handle food in order to minimize food loss and waste (Vermesan et al., 2011). However, there are a number of technical (lack of technical expertise), financial (high capital costs involved in setting up a system of sensors, for example), social (issues regarding data privacy and sharing), operational (lack of standards and communication protocols), educational (educating food service providers to switch from a well-established system to a newer system; educating the public), and governmental (lack of standardized regulations) challenges that work against the use of IoT in the food industry (Aamer et al., 2021; Ahmadzadeh et al., 2023).

Additional artificial intelligence (AI)-based applications to minimize food waste include the development of AI-based dynamic pricing to reduce the selling price of food closer to the “Sell by” or “Use by” date, or rotating food out based on a “first expired, first out” system, rather than the prevalent “first in, first out” system.

## 6. Discussion

According to the United Nations Sustainable Development Goals report, an estimated 13.3% of the food produced in the world was lost along the supply chain in 2020 (post-harvest, pre-retail) along with an estimated 17% of food available to customers [post-retail; United Nations (UN), 2023b]. Food loss and waste have many broad-ranging implications. However, formulating effective policies and strategies to reduce food loss and waste has proven to be difficult, since this necessitates comprehensive information regarding the how, why, how much, and where along the supply chain (pre-retail) and beyond (retail and post-retail) food is lost or wasted [United Nations (UN), 2023c]. Under such circumstances, a specific focus on spoilage-related food loss and waste has proven to be difficult. This is particularly true for food lost in the supply chain, since data pertaining to food loss during the production and processing stages can be attributed to a number of causes, such as compliance with internal specifications or regulatory requirements, which may or may not be related to food being unfit for human consumption. In essence, while most sustainability and food waste mitigation initiatives aimed toward the food supply chain (i.e., pre-retail) focus on methods to re-use or re-purpose foods that are discarded due to physical deformities, packaging or cosmetic defects, and non-compliance of the product with regulatory, legislative, seller, or consumer specifications, research into the means to prevent food loss caused by microbiological or chemical concerns remains lacking. This is also reflected in the overall lack of knowledge about the actual proportion of food being wasted due to microbiological concerns. Current research into preventing microbiological spoilage and, by extension, food loss, in the supply chain has primarily focused on the development of novel packaging technologies and monitoring of temperature and other indicators of food quality (such as carbon dioxide, enzymatic reactions, or pH levels). However, the food supply chain is yet to take an active *preventative* approach to minimizing spoilage-related food loss. This is where widespread adoption of a standardized QMSRA framework would be helpful, as it could provide a scientific basis for developing strategies to more effectively manage food spoilage, extend shelf life and limit food waste at a global scale. This, in combination with increased adoption of portable and wireless sensors, which typically monitor factors impacting, or indicators of, food spoilage, such as ambient temperatures and the atmospheric content, would help in minimizing uncertainty and variability in spoilage risk calculations, thereby improving shelf life predictions along the supply chain. Although the use of such sensors is currently limited because of poor battery life, high capital costs, data transmission issues (particularly in high moisture foods (Jedermann et al., 2014b)), generation of false positive or negative results, potential human health risk from the leakage of chemicals, as well as the knowledge that the presence of certain target metabolites alone need not be indicative of poor quality (which in turn would contribute to additional food and resource waste; Poyatos-Racionero et al., 2018), current research into the development of alternative sensors that use natural indicator compounds proves to be promising in allowing for more widespread adoption in the near future. In combination, these techniques and technologies have the potential to significantly reduce uncertainty regarding product freshness and viability, minimize the need for destructive testing, and overall reduce food loss and waste both in the pre-retail supply chain level and in the retail and food service provider stages.

From a consumer perspective, food waste appears to be largely driven by the perceived level of freshness and safety of a food product, which in turn are influenced greatly by date labels (Li et al., 2020). However, the US Food and Drug Administration (FDA) mentioned that “most date labels are not based on exact science” (Patra et al., 2022). A great number of studies support this opinion. Laboratory tests of milk, pasta, mayonnaise, and jam have found that products remain safe to eat up to over six months after the ‘best before’ date from the perspective of microbiological safety, with the texture, color, and sensory quality only decreasing slightly after one month for pasteurized milk and mayonnaise and after three months for pasta and jam (Zielińska et al., 2020). Thus, with the lack of an accurate way to determine if the food is still fresh, consumers rely blindly on date labels, which in turn contributes to a large percentage of food being wasted at the consumer level. This highlights the importance of intelligent, easy-to-comprehend freshness indicators that can remove the guesswork out of deciding if a food product can still be consumed. Particularly, indicators that can communicate the shelf life of a food product *after* it has been opened could potentially significantly reduce the incidence of food waste at the consumer level. A majority of such smart indicators that are currently available (or are being developed) detect noticeable changes in the carbon dioxide levels or those of metabolites associated with degradation in food packages, which can occur due to microbial spoilage, fermentation, or ripening (such as the After Opening labels by Insignia Technologies and the RipeSense® sensor label). However, despite interest and capital being devoted to the development of these technologies, widespread commercial application and implementation remains rare.

In conclusion, despite the abundance of scientific literature and emerging technologies focused on minimizing and reducing food waste across the supply chain up to the consumer level, they may not be implementable for some time, due to process-related or regulatory concerns, or sustainable, due to the high costs involved. For example, intelligent packaging systems with chemical-based indicator labeling technology that are directly in contact with food would be classified as indirect additives by the U.S. Code of Federal Regulations (CFR Title 21 § 174.5), while novel technologies that directly interact with food items to minimize spoilage and spoilage-related activities to increase shelf life would be classified as direct additives. Moreover, manufacturers would additionally need to be aware of the by-products of chemical decomposition and impurities resulting from chemical activity over time. These, in turn, would necessitate stringent safety assessments to determine the risk of dietary exposure to these chemicals. Alternatively, manufacturers must increase focus on developing applications for commercial use that is based on relatively safe chemical reactions, such as Maillard browning. On the other end of the spectrum, there is currently no regulatory or legislative impetus for increased adoption of QMSRA by the food industry, despite the interest shown by researchers regarding its potential and the proven track record of its sister QMRA framework to quantify the risk posed by pathogenic microorganisms to human health. Moreover, due to the novelty of this concept in the field of microbial spoilage, the availability of quantitative data needed to perform these assessments is scarce.

Studies have shown that social innovation-based interventions may be the answer to sustainably reduce food waste in the long run. These include the development of new regulations regarding the alternative use of misbranded or “economically adulterated,” but otherwise safe, products. For example, the United States Department of Agriculture (USDA) has enacted new regulations that allow for the donation of misbranded, but unadulterated and otherwise safe to eat, animal meat products, as well as otherwise fresh and safe imported produce items that do not meet the regulatory specifications set by the USDA to charitable organizations [United States Department of Agriculture (USDA), 2023]. At a processing level, methods to minimize food spoilage and waste may not be universally applicable to all companies. However, the utilization of some basic methods could help substantially minimize the losses encountered due to food waste – losses due to inadvertent circumstances such as power blackouts and equipment defects, which could endanger product quality and safety, could be prevented by the use of emergency power supplies and extensive plant maintenance (Raak et al., 2017). On the other hand, in cases where such losses are inevitable, minimally spoiled food can be re-purposed (milk → whey, tomatoes → sauce, ketchup, or marinara, fruits → jam or filling for sweet confections, among others) while food with major spoilage or safety concerns can be used in the production of alternative sources of fuel. Finally, consumer perception of food quality and safety, particularly when regarding date labels and suboptimal foods, must be addressed on a global scale in order to minimize discarding of “expired but acceptable” foods.

## Author contributions

SK, SF, DP, and AP contributed to the conception and design of the study. SK and SF curated and compiled all relevant data. SK wrote the first draft of the manuscript with help from SF. DP and AP reviewed and edited the manuscript. AP supervised the study. All authors contributed to manuscript revision, read, and approved the submitted version.

## Conflict of interest

The authors declare that the research was conducted in the absence of any commercial or financial relationships that could be construed as a potential conflict of interest.

## Publisher’s note

All claims expressed in this article are solely those of the authors and do not necessarily represent those of their affiliated organizations, or those of the publisher, the editors and the reviewers. Any product that may be evaluated in this article, or claim that may be made by its manufacturer, is not guaranteed or endorsed by the publisher.

## Footnotes

^1^It is important to note that, except for infant formula, the U.S. Food and Drug Administration does not mandate the “Use by” date labels for any food products that fall under its purview. Similarly, the U. S. Department of Agriculture only requires a “pack date” for poultry products and thermally processed, commercially sterile products to help identify product lots and facilitate trace-back activities in the event of an outbreak of foodborne illness [9 Code of Federal Regulations 381.126 and 431.2(e)].
